# Adaptive immune changes in colorectal cancer: a focus on T and B cell activation genes

**DOI:** 10.1007/s12672-025-02794-8

**Published:** 2025-06-08

**Authors:** Mona Rady, Albert Ashraf, Hesham Abdelaziz, Mohamed El-Azizi

**Affiliations:** 1 Microbiology, Immunology and Biotechnology Department, Faculty of Pharmacy and Biotechnology, the German University in Cairo, Cairo, Egypt; 2 Faculty of Biotechnology, German International University, New Administrative Capital, Cairo, Egypt; 3 National Cancer Institute, Department of Clinical Pathology, Cairo University, Cairo, Egypt

## Abstract

Colorectal cancer (CRC) ranks as the third most common cancer globally and is the second leading cause of cancer-related deaths, following lung cancer. Despite the immune system’s capacity for tumor surveillance and elimination, CRC tumors can evade immune responses through complex mechanisms, ultimately escaping immune detection. T and B lymphocytes play a critical role in adaptive immunity against tumors, with T cells, particularly CD8^+^ cytotoxic T cells, driving tumor elimination. Additionally, B cells contribute by producing tumor-specific antibodies, including various immunoglobulin G (IgG) subclasses that participate in immune modulation. However, the effectiveness of adaptive immunity in CRC is often limited due to tumor-driven immunosuppression. This study investigates the expression of T and B cell activation genes in peripheral blood mononuclear cells (PBMCs) isolated from CRC patients. A panel of 84 genes involved in T and B cell activation was analyzed to assess changes in expression using RT^2^ QPCR arrays. Additionally, we measured serum levels of the four IgG subclasses (IgG1, IgG2, IgG3, and IgG4) in CRC patients to explore possible alterations in humoral immunity. Compared to healthy controls, 5 genes were found to be downregulated in PBMCs of all CRC patients’ groups; *CCL3* (fold regulation − 6.36), *IL6* (fold regulation − 12.46), *CSF2* (fold regulation − 7.50), *CXCR3* (fold regulation − 3.01), and *TNFSF14* (fold regulation − 4.90). Moreover, 13 genes were upregulated in PBMCs of all CRC patients’ groups; *CCR3* (fold regulation 59.21), *CD2* (fold regulation 3.07), *CD27* (fold regulation 6.39), *CD3G* (fold regulation 4.15), *CD8B* (fold regulation 3.25), *FAS* (fold regulation 3.94), *IL10* (fold regulation 39), *IL18R1* (fold regulation 82.39), *IL5* (fold regulation 20.4), *LAG3* (fold regulation 19.88), *MAP3K7* (fold regulation 4.07), *TLR1* (fold regulation 6.45), and *TLR6* (fold regulation 18.87). The serum levels of the four IgG subclasses were however statistically insignificant in CRC patients compared to healthy controls. Our findings provide insights into the adaptive immune dysfunction in CRC, offering a detailed profile of gene expression changes associated with T and B cell activation and antibody production. Understanding these dysregulations may enhance the development of targeted immunotherapies, potentially improving outcomes for CRC patients through more personalized immunomodulatory approaches.

**Supplementary Information:**

The online version contains supplementary material available at 10.1007/s12672-025-02794-8.

## Introduction

CRC resides at third place for being the most common type of cancer after breast and lung cancers in terms of incidences worldwide and holds second place for causing most mortalities worldwide after lung cancer according to GLOBOCAN 2020 [1]. According to GLOBOCAN 2020, out of the 185 countries, Egypt took 46th place in incidence rates of CRC. Total incidence rates of CRC for both sexes were reported to be 5,231 cases. Mortality wise, Egypt also held 46th place out of 185 countries listed in the statistics of GLOBOCAN 2020 for CRC with total mortality cases of 2,852 cases for both sexes [1].

Immune evasion is one of the hallmarks of cancer [2]. While strong evidence supports the concept of immunosurveillance and immunoediting in CRC, the immune system, particularly the adaptive one fails in controlling tumor growth, because of strong immune-evasion mechanisms developed by the tumor [3–5]. Studies have shown that CRC is associated with downregulation of MHC-I molecules and the antigen processing machinery [4, 6]. Moreover, recent evidence suggested that in spite of the pronounced infiltration of CRCs with effector T cells, this was counterbalanced by an upregulation of immune checkpoint molecules [7]. Using whole transcriptome RNA-seq analysis of PBMCs isolated from CRC patients, Shaath et al. revealed altered immune signature and identified immune response-related genes to be enriched in CRC patients. Among the upregulated genes in PBMCs in CRC patients are *CXCL2, IL8, CCL7, CXCL3, IL10* and *CCL3*. Among the downregulated genes in PBMCs in CRC patients are *TLR7, TLR5, TLR10, TLR8, TNFSF10* and *CD79B* [8]. Similarly, Nichita et al. detected unique gene expression signature in PBMCs isolated from CRC patients [9]. Using high throughput real-time PCR Ciarloni et al. reported discriminate gene expression signature of 29 genes in PBMCs from CRC patients [10]. Also recently, using pan-cancer scRNA-seq analysis, Zhang et al. characterized the immune cell landscape in PBMCs from various cancer types, including CRC. They identified distinct immune cell populations and gene expression patterns associated with CRC, providing valuable insights into the tumor microenvironment [11]. Similarly previous studies documented unique immune response-related signature in PBMCs isolated from breast [12], pulmonary [13], renal [14, 15], bladder [16] as well as digestive [17] cancer patients.

IgG, the predominant antibody isotype, comprises four subclasses (IgG1, IgG2, IgG3, IgG4), each with distinct functions and flexibility. IgG4, representing only 4% of total IgG, exhibits unique “Fab arm exchange” capabilities, enabling hybrid molecule formation [18]. In fact, evidence exists that there is IgG4 bias in some tumors [19]. Examples of such tumors are melanoma [20–22], pancreatic cancer [23], glioblastoma [24], and extrahepatic cholangiocarcinoma [25, 26]. In CRC, high IgG4 synergizes with macrophages in shaping an immunosuppressive microenvironment and alters anti-tumor effector cell functions [27].

In recent years, several immunotherapeutic approaches have been tested in CRC. Of these, boosting the immune system with cytokines, immune checkpoint inhibitors, cancer vaccines, and adoptive cell therapy proved to be the most promising strategies [28]. The present study aims at shedding some light on the change in gene expression of a focused panel of genes responsible for T and B-cell activation in PBMCs isolated from patients with CRC. This study also aims at measuring serum levels of the four IgG subclasses in CRC patients. Therefore, this study provides a comprehensive assessment of the adaptive immune response in CRC. Comprehension of antitumor immune response in patients with CRC may allow the effective application of the different approaches of immunotherapy for the treatment of CRC in the near future.

## Materials and methods

### Patients, healthy donors and peripheral blood samples

A total of 75 peripheral blood samples were collected from patients with confirmed diagnosis of CRC who are admitted at the National Cancer Institute (NCI)-Cairo University. All patients received and signed an informed consent according to the declaration of Helsinki. Peripheral blood samples were collected in 2 lithium heparin vacutainers (for PBMCs isolation) and 1 serum vacutainers (for IgG subclass measurements). The study was approved by the local ethical committees of the NCI and the German University in Cairo (GUC). All peripheral blood was sampled from patients first admitted to the NCI-Cairo, with confirmed diagnosis of CRC before administration of any chemotherapy, radiotherapy, or immunotherapy. Samples with very suboptimal RNA concertation and/or purity were excluded. This reduced the total number of samples used in this study from 75 to 19 samples in total, of which were 9 females and 10 males. The control group consisted of 12 males and 8 females, selected based on the criteria of being healthy individuals with no history of chronic or acute diseases, chronic diseases, or current or recent infections at the time of sample collection. Samples were collected following the same protocol as for CRC patients: two lithium heparin tubes were used for RNA isolation and gene expression analysis via PCR, and one serum tube was used for IgG subclass assessment via ELISA. Table 1 shows the characteristics of CRC patients and healthy donors included in this study after exclusion of samples of suboptimal RNA yield and/or purity.

**Table 1:** Characteristics of CRC patients and healthy donors

	HD	CRC patients
**Number**	20	19
Age (median)*	32.5	44.8
**Gender (male, female)**	12,8	(10,9)
**TNM stage**		
Stage I		0
Stage II		5
Stage III		3
Stage IV		3
**Anorectal cancer**		3
**Rectal cancer**		5
**Histological grade**		
Poorly differentiated		9
Well/Moderately differentiated		10

*HD* Healthy donors, *CRC* Colorectal cancer

^*^Median range

### PBMCs isolation

PBMCs were isolated by density gradient centrifugation by layering the blood on Histopaque^®^-1077 (Sigma-Aldrich’s) following the standard separation protocol recommended by the manufacturer. PBMCs were isolated within 4 h of blood samples collection.

### Total RNA extraction

PBMCs were lysed immediately after isolation in RLT buffer (Qiagen) supplemented with β-mercaptoethanol (Sigma) and stored at − 80 °C until RNA extraction is initiated. RNA was extracted using Qiagen’s RNEasy^®^ Mini Kit RNA columns according to the manufacturer’s directions. RNA concentration and purity took place using Thermo Fisher Nanodrop^™^ 2000 instrument and Thermo Software IQ software program at λ_260_ nm and λ_280_ nm. The yield and the purity of the RNA were calculated and determined automatically via the software.

### cDNA synthesis

Total RNA was used as a templated for cDNA synthesis using RT^2^ first strand cDNA kit (Qiagen) that includes a DNAase treatment step, reducing the likelihood of amplifying genomic DNA. Patient total RNA samples were grouped into 5 groups; colon cancer stage II, colon cancer stage III, colon cancer stage IV, anorectal cancer and rectal cancer. To ensure comprehensive analysis of gene expression profiles across cancer patients, RNA samples from patients representing all five groups (colon cancer stage II, colon cancer stage III, colon cancer stage IV, anorectal cancer and rectal cancer) were pooled for use in QPCR arrays, allowing for a robust comparison of target gene expression patterns while minimizing individual patient variability. Similarly, the total RNA from healthy controls was pooled to minimize individual donor variability. Reverse transcription was done using 1 μg total RNA in a final reaction volume of 20 μl and according to the manufacturer’s directions.

### Real-time qPCR using RT^2^ Profiler PCR arrays

The reverse transcription mix was completed to 111 μl. The PCR reaction mixes were prepared in RT^2^ PCR Arrays Loading Reservoir (Qiagen). The product of cDNA synthesis reaction of a volume of 102 μl was added to 1350 μl of RT^2^ SYBR Green ROX qPCR Mastermix (Qiagen), completed to a final reaction volume of 2700 μl with nuclease free water. The PCR components mix were dispensed into the RT^2^ Profiler™ PCR Arrays (Human T-Cell & B-Cell Activation) (catalogue # 330231, GeneGlobe ID: PAHS-053Z). QPCR was performed using Applied Biosystems™ StepOnePlus^™^ Real-Time PCR cycler. The following thermal profile was used: 95 °C for 10 min, followed by 40 cycles of 95 °C for 15 s and 60 °C for 1 min. A dissociation curve was run at the end of each PCR run using the following thermal profile: 95 °C for 1 min, 55 °C for 30 s, 95 °C for 30 s to test for the specificity of each assay.

### RT^2^ profiler data analysis

The data analysis was performed with StepOnePlus software version 3.2 (Applied Biosystems). The Cycle threshold (Ct) values were generated individually, for each cancer patients’ group and healthy donors’ group for each target gene. The Ct values for all wells were exported to a blank Excel spread sheet. Data analysis was conducted using QIAGEN’S GeneGlobe Data Analysis Center available at https://geneglobe.qiagen.com/eg/analyze. The five reference genes *ACTB*, *B2M*, *RPLP0*, *GAPDH* and *HPRT1* were used to normalize relative expression ratios by calculating the 2^(−ΔΔCt)^ for each gene in the plate [29].

### IgG subclass quantification

Serum samples from cancer patients and health donors were used for measuring the levels of IgG subclasses using commercial Human IgG subclass ELISA kit (ThermoFisher Scientific, catalogue# 991000. Serum samples were pooled into 5 cancer patients’ groups and 1 healthy donor group similarly to minimize individual patient/donor variability. Quantification was done using TECAN’s Infinite^®^ F50 spectrophotometer set at λ_450_ nm absorbance, and data were analyzed using TECAN’s Magellan^™^ data analysis software.

## Results

### Eighteen T cell and B cell activation genes are differentially expressed in PBMCs of CRC patients

Total RNA was extracted from PBMCs isolated from CRC patients and reverse transcribed into cDNA. The expression of 84 T and B cell activation genes was done using RT^2^ QPCR arrays. The RT^2^ QPCR arrays analysis revealed a total of 18 T cell and B cell activation genes to be differentially expressed in all CRC patients’ groups analyzed. Table 2 shows a list of all T and B cell activation genes up and down-regulated with fold regulation >|2|.

**Table 2:** Differentially expressed T and B cell activation genes in PBMCs of CRC patients

Unigene	Refseq	Symbol	Description	Fold Regulation (geometric mean)	*P* value
Hs.514107	NM_002983	CCL3	Chemokine (C–C motif) ligand 3	− 6.36	0.0054
Hs.506190	NM_001837	CCR3	Chemokine (C–C motif) receptor 3	59.21	< 0.0001
Hs.523500	NM_001767	CD2	CD2 molecule	3.07	0.0014
Hs.355307	NM_001242	CD27	CD27 molecule	6.39	0.0181
Hs.2259	NM_000073	CD3G	CD3g molecule, gamma (CD3-TCR complex)	4.14	0.0064
Hs.405667	NM_004931	CD8B	CD8b molecule	3.25	0.0017
Hs.667309	NM_000043	FAS	Fas (TNF receptor superfamily, member 6)	3.94	0.0020
Hs.193717	NM_000572	IL10	Interleukin 10	36.00	0.0002
Hs.469521	NM_003855	IL18R1	Interleukin 18 receptor 1	82.39	< 0.0001
Hs.2247	NM_000879	IL5	Interleukin 5 (colony-stimulating factor, eosinophil)	20.40	0.0012
Hs.654458	NM_000600	IL6	Interleukin 6 (interferon, beta 2)	− 12.46	0.0203
Hs.409523	NM_002286	LAG3	Lymphocyte-activation gene 3	19.88	< 0.0001
Hs.594838	NM_003188	MAP3K7	Mitogen-activated protein kinase kinase kinase 7	4.07	0.0029
Hs.654532	NM_003263	TLR1	Toll-like receptor 1	6.45	< 0.0001
Hs.575090	NM_006068	TLR6	Toll-like receptor 6	18.87	0.0002
Hs.1349	NM_000758	CSF2	Colony stimulating factor 2 (granulocyte–macrophage)	− 7.50	0.0074
Hs.198252	NM_001504	CXCR3	Chemokine (C-X-C motif) receptor 3	− 3.01	0.0017
Hs.129708	NM_003807	TNFSF14	Tumor necrosis factor (ligand) superfamily, member 14	− 4.90	0.0031

**Fig. 1:**
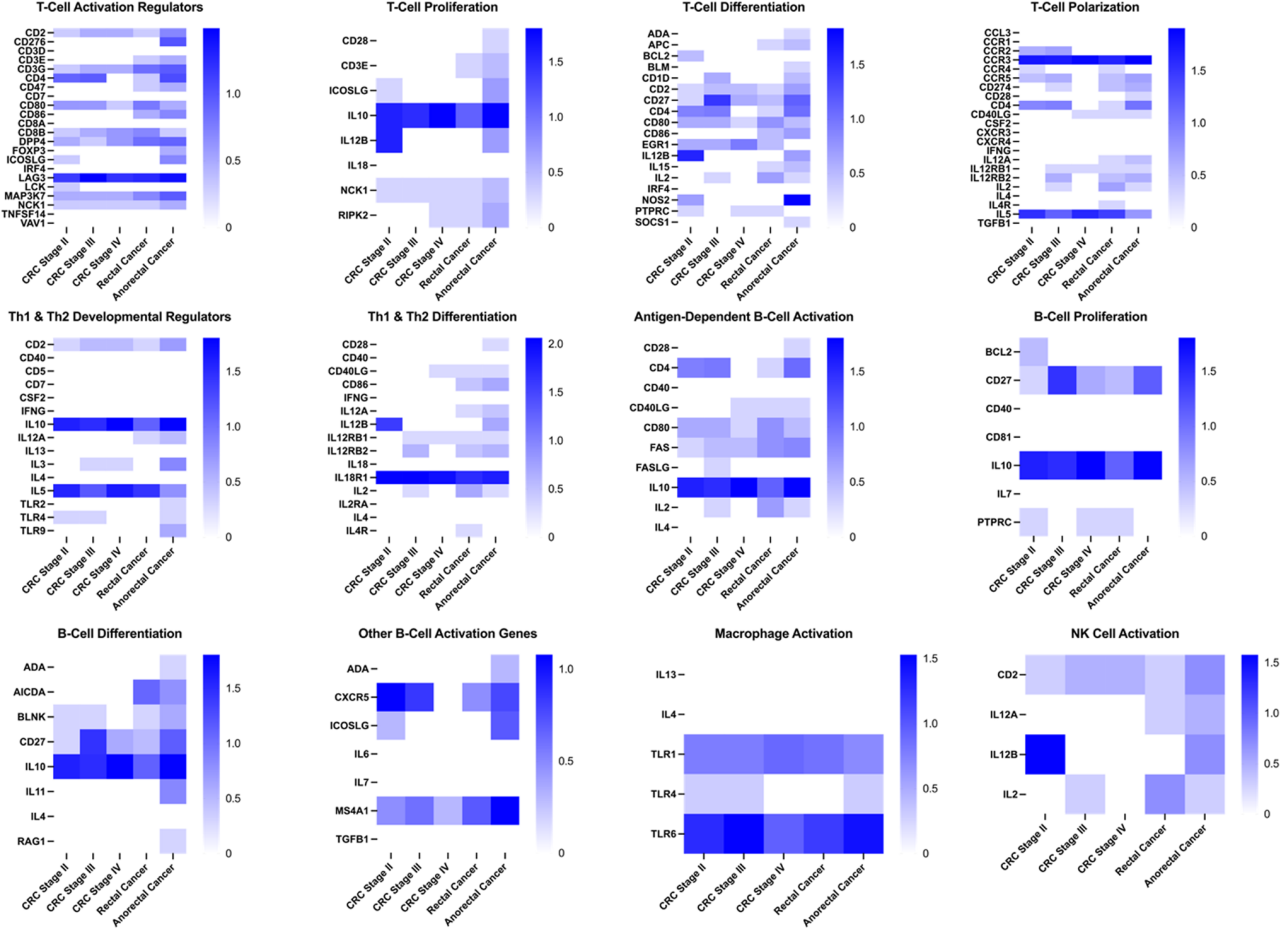
Heatmap of relative quantification of T and B cell activation genes as well as macrophages and NK cell activation genes in PBMCs isolated from CRC patients and healthy controls. The columns represent CRC patients and the rows represent the different T cell and B cell activation genes as well as macrophages and NK cell activation genes. The color gradient blue-white represents relative level of gene expression, indicating up-down regulation, respectively, where blue indicates upregulation and white indicates downregulation (log fold change). Data represent log relative expression ratios (log fold change). Relative expression ratio (2^(−ΔΔCT)^) is the normalized gene expression (2^(−ΔCT)^) in the CRC samples divided the normalized gene expression (2^(−ΔCT)^) in the healthy control samples. The heatmaps were generated using the GraphPad Prism version 9.5.1 for macOS

Figure 1 presents the heatmaps of the relative quantification of T cell and B cell activation genes, as well as macrophages and NK cell activation genes included in the RT^2^ QPCR arrays in PBMCs of CRC patients compared to healthy controls. Columns represent the CRC patients, and rows represent the differentially expressed genes. The heatmaps illustrate the expression levels of different T cell and B cell activation genes as well as macrophages and NK cell activation genes, with blue-white color gradients indicating upregulation and downregulation, respectively. The heatmaps were generated using the GraphPad Prism version 9.5.1 for macOS. Supplementary Fig. 1 shows a scatter plot comparing the average fold regulation of 84 genes between the CRC patients and healthy control groups.

**Fig. 2:**
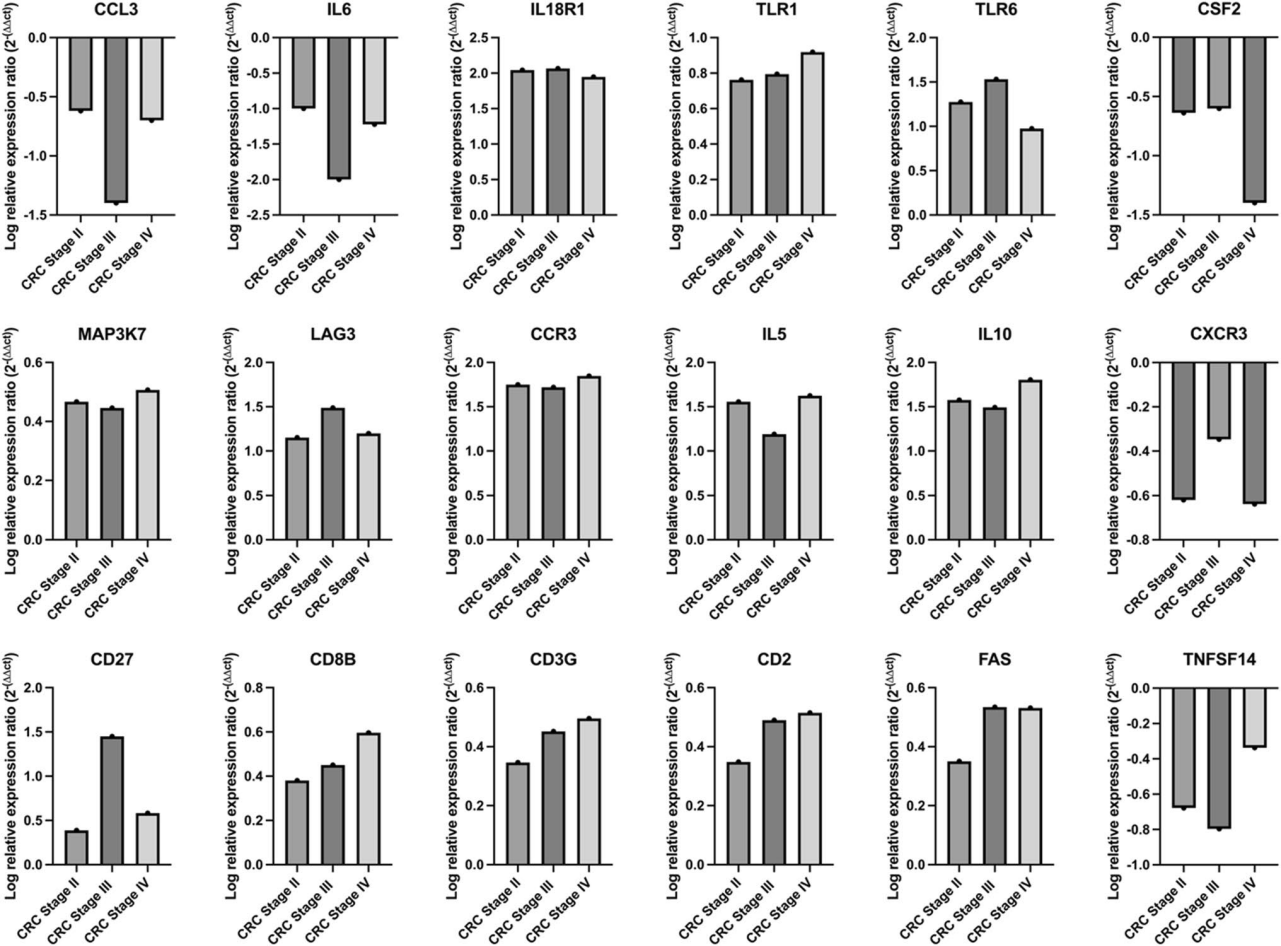
Relative mRNA expression of genes differentially expressed in stages II, III, and IV of CRC. Relative expression ratio (2^(−ΔΔCT)^) is the normalized gene expression (2^^(−ΔCT)^) in the CRC samples divided the normalized gene expression (2^(−ΔCT)^) in the healthy control samples. Data shows log relative expression ratios with log relative expression ratios > 0 represent upregulated genes and log relative expression ratios < 0 represent down regulated genes

The relative mRNA expression levels of eighteen differentially expressed across all CRC cancer patient groups were analyzed across stages II, III, and IV of CRC compared to healthy controls (Fig. 2). Genes with log relative expression ratios greater than 0 were identified as upregulated, while those with log relative expression ratios less than 0 were considered downregulated. The analysis revealed distinct patterns of gene regulation correlating with CRC progression, highlighting specific genes that may play a pivotal role in advancing disease stages. These findings provide valuable insights into the molecular mechanisms underlying CRC disease progression. Supplementary Figs. 2 and 3 show gene interaction network of downregulated and upregulated genes, respectively in PBMCs from patients with CRC identified using GeneMANIA tool.

### IgG subclasses expression levels are unaltered in CRC patients

**Fig. 3:**
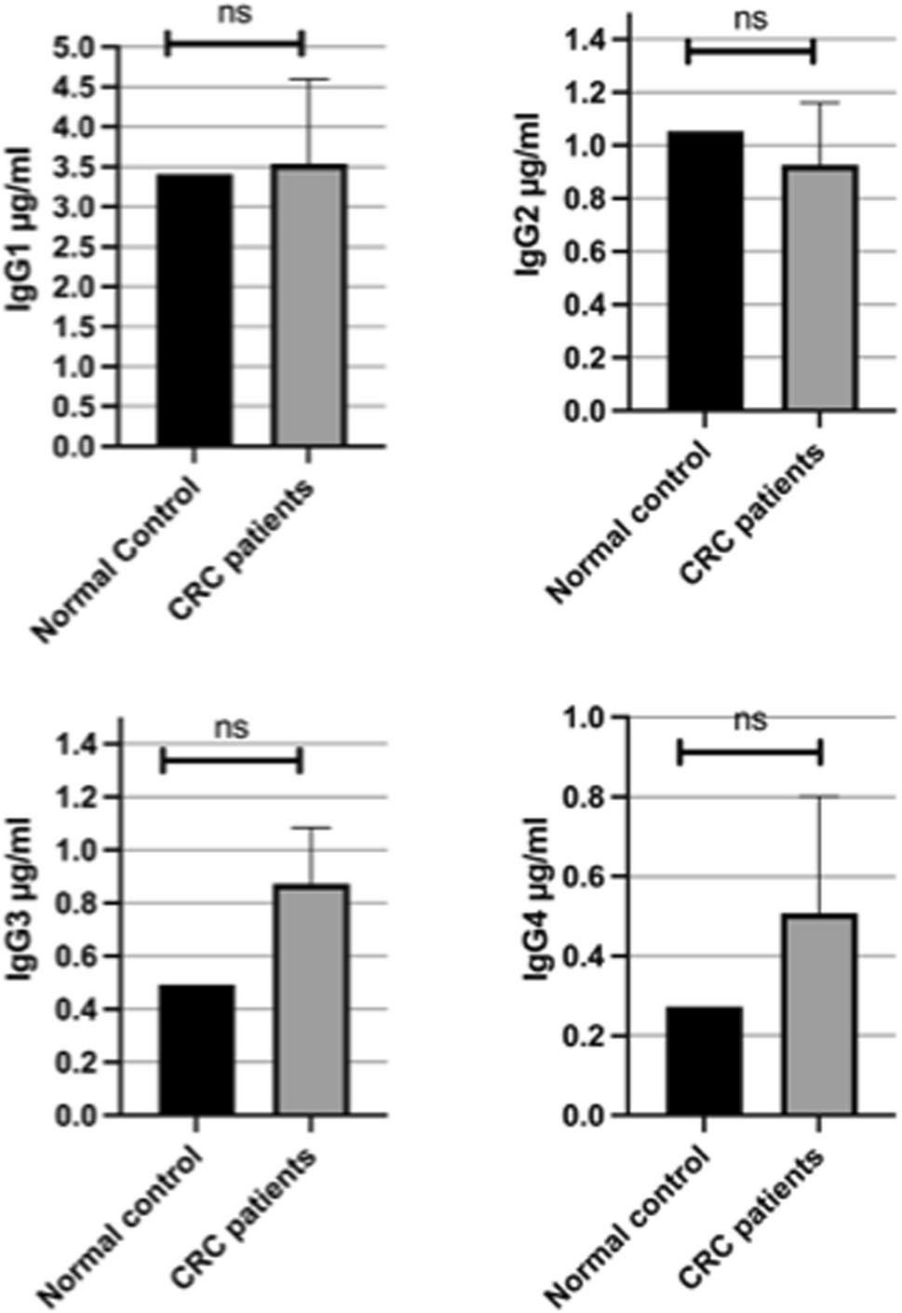
Expression analysis of IgG subclasses; IgG1, IgG2, IgG3 and IgG4 in serum samples of CRC patients compared to healthy controls. Data represent the average serum concentration of IgG subclasses of 19 CRC patients as measured by ELISA. Serum levels of the four IgG subclasses show nonsignificant difference compared to healthy controls

Serum levels of IgG subclasses were evaluated using ELISA in CRC patients and healthy controls. No statistically significant differences were found for any of the subclasses. IgG1 concentrations were comparable between CRC patients and controls (*P* = 0.9143). For IgG2, a slight decrease was noted in CRC patients, but this did not reach statistical significance (*P* = 0.6467). Similarly, IgG3 levels showed a non-significant increase in CRC patients (*P* = 0.1749). IgG4 levels were also elevated in CRC patients but remained statistically insignificant compared to controls (*P* = 0.5066). These findings suggest no marked alteration in systemic IgG subclass levels in CRC under the current study conditions. Figure 3 shows the expression analysis of IgG subclasses by measuring IgG subclasses concentrations in serum samples of CRC patients compared to healthy controls.

## Discussion

PBMCs represent a valuable tool in immunological research providing a focused perspective on the body’s immune activities, because they include various immune cell populations, such as lymphocytes (T cells, B cells, and NK cells), monocytes, and dendritic cells. [30]. Here we investigated the expression of T and B cell activation genes in PBMCs isolated from CRC patients. A panel of 84 genes involved in T and B cell activation was analyzed to assess changes in expression using RT^2^ QPCR arrays. Additionally, we measured serum levels of the four IgG subclasses (IgG1, IgG2, IgG3, and IgG4) in CRC patients to explore possible alterations in humoral immunity.

Compared to healthy controls, five genes were found to be downregulated in PBMCs of all CRC patients’ groups; *CCL3, IL6, CSF2, CXCR3, and TNFSF14*. Moreover, 13 genes were upregulated in PBMCs of all CRC patients’ groups; *CCR3, CD2, CD27, CD3G, CD8B, FAS, IL10, IL18R, IL5, LAG3, MAP3K7, TLR1* and *TLR6*. The serum levels of the four IgG subclasses were however statistically insignificant in CRC patients compared to healthy controls.

*CCL3* or C–C Motif Chemokine Ligand 3 is among the downregulated genes in PBMCs of all CRC samples. The encoded protein is a monokine with inflammatory and chemokinetic, also known as macrophage inflammatory protein 1 alpha, plays a role in inflammatory responses through binding to the receptors CCR1, CCR4 and CCR5. CCL3 plays an important role in recruitment of CD8^+^ T cells, NK cells and macrophages, particularly proinflammatory (M1) to the tumor microenvironment [31]. Therefore, the decreased expression of *CCL3* in the immune system of CRC patients would lead to impaired immune surveillance and tumor cell killing. Moreover, reduced expression of *CCL3* has the potential to contribute to the creation of an immunosuppressive tumor microenvironment leading to tumor progression. This is because reduced CCL3 expression would favor the pro-tumor M2 macrophages [32], which suppress immune responses and promote tumor growth and metastasis. Our finding that *IL10* is upregulated in PBMCs from CRC patients supports this conclusion as IL10 is secreted from alternatively activated M2 macrophages [33]. Since CCL3 can enhance the therapeutic effect of immune checkpoint inhibitors (like PD-1 inhibitors) [34], a reduction in CCL3 expression in PBMCs of CRC patients implies that patients could respond less effectively to immunotherapies that depend on strong immune cell infiltration. Taken together, reduced *CCL3* levels in PBMCs a reflects a systemic immune dysregulation, reduced immune cell recruitment to the tumor, potentially diminishing the efficacy of the anti-tumor activity against CRC cells promoting tumor growth and metastasis [35].

IL6 is a proinflammatory cytokine that plays also a role in B cell maturation. Therefore, the reduced expression of *IL6* in PBMCs isolated from CRC patients indicates an immunosuppressive environment created by the tumor allowing tumors to evade immune detection. This suggests that the tumor microenvironment itself can modulate immune responses by dampening IL-6 production in circulating immune cells like PBMCs. While IL6 plays two opposing roles in the tumor microenvironment, accumulating evidence establishes IL-6 as a key player in the activation, proliferation and survival of T cells and NK cells during active immune responses [36, 37]. This might suggest that CRC patients have a reduced capacity to mount an effective immune response due to lower IL-6 levels in circulating PBMCs.

Similarly, the downregulation of *CSF2*, *CXCR3*, and *TNFSF14* in PBMCs from CRC patients suggests a potential impairment in immune activation and anti-tumor responses. CSF2 (granulocyte–macrophage colony-stimulating factor) plays a key role in myeloid cell differentiation and immune stimulation [38]. In fact, CSF2 behaves as a double-edged sword in cancer by enhancing both anti- and pro-tumorigenic immune cells depending on its expression, cancer type, and tumor immune microenvironment [39]. The reduced expression of CSF2 may, however, contribute to diminished antigen presentation and immunosuppression in CRC. CXCR3, a chemokine receptor essential for T cell and NK cell migration to tumor sites, is closely associated with effector immune responses [40]. Its downregulation may indicate impaired lymphocyte trafficking and immune evasion by CRC cells. Similarly, TNFSF14 (also known as LIGHT), a TNF superfamily member involved in co-stimulation and lymphoid tissue organization, is critical for lymphocyte activation [41]. Its reduced expression may reflect tumor-induced suppression of the immune microenvironment, as LIGHT signaling has been linked to anti-tumor immunity in gastrointestinal cancers [42]. Collectively, these findings suggest systemic immune dysregulation in CRC, potentially facilitating tumor progression by dampening pro-inflammatory signaling and cytotoxic lymphocyte recruitment.

*IL18R1* is among the genes that are consistently upregulated in PBMCs of patients with CRC. It is well known that expression of this receptor is induced by IFN-α and IL-12 in both NK cells and T cells. Thus, overexpression of IL18R1 on NK cells and T cells could lead to amplified IL-18 signaling, which is known to activate various immune cells, including T cells, NK cells, and macrophages. Although this could contribute to a stronger anti-tumor immune response, it can also indicate strong chronic inflammation that can also create a favorable environment for tumor growth and metastasis [43]. In fact, IL-18 signaling might contribute to this by promoting angiogenesis, tissue remodeling, and immunosuppression [44]. In fact, IL-18 has been implicated in regulating immune checkpoints, such as PD-1/PD-L1 [45, 46]. Stimulation of IL-18 signaling was shown to up regulate the expression of PD-1 by conventional NK cells converting them into a subset with immunosuppressive characteristics [45, 46]. Overexpression of IL18R1 might influence the PD-1/PD-L1 inhibitory pathways, potentially leading to immune evasion by cancer cells. Overexpression of *TLR1, TLR6* and *MAP3K7* also indicate a state of chronic inflammation associated with CRC.

*LAG3* or lymphocyte activation gene-3 is an inhibitory immune checkpoint that exerts inhibitory effect on various lymphocytes such as T cells, Tregs, B cells, invariant NKT cells and NK cells [47–49]. LAG3 which is closely related to CD4, binds to MHC molecule class II on APCs with high affinity [50]. Elevated expression of *LAG3* in PBMCs suggests that LAG3 could be contributing to the immune evasion mechanisms employed by the tumor. This upregulation might reflect a state of immune exhaustion or suppression. It was also previously shown that LAG-3^+^ NK cells had impaired cytotoxic capacity and are resistant to K562 target cell stimulation [51]. LAG3 was shown to reduce T cell proliferation and cytokine secretion, as evidenced by enhanced T cell activation following blocking of LAG3 by an antibody [52]. Moreover, it was shown that chronic tumor antigen stimulation leads to persistent overexpression of LAG3 on CD8^+^ tumor antigen-specific T cells, leading to their functional exhaustion [53].

The protein encoded by *CCR3* is a G protein-coupled receptor for C–C type chemokines. Chemokine ligands for this receptor includes CCL11, CCL26, CCL7, CCL13, and CCL5 [54]. CCR3 is expressed exclusively on TH-2 cells [55]. Here we show that *CCR3* was overexpressed in PBMCs isolated from CRC patients. Tian et.al (2016) showed that CCR3, was overexpressed and that CCL11/CCR3 signaling promoted the proliferation, migration and invasion of glioblastoma cells [56]. Being exclusively expressed on TH2 cells, its over expression in PBMCs from CRC patients indicates a shift towards a TH2-dominated immune response in CRC patients. TH2 cells can in turn suppress anti-tumor immune responses mediated by TH1 cells, cytotoxic T cells, and NK cells. This could allow the tumor to escape immune surveillance and enhance tumor progression. CCR3 signaling have been implicated in processes like angiogenesis, tumor cell migration, and metastasis [57]. Consistent with this is our finding that *IL5* is upregulated in PBMCs from CRC patients suggesting a bias towards TH2 response. *CCR3* upregulation in PBMCs might reflect its involvement in promoting metastatic spread or local invasion of tumors. Upregulation of *CCR3* could also indicate an enhanced inflammatory response, where the tumor secretes chemokines that recruit immune cells expressing CCR3. This could represent one strategy that tumors employ to manipulate the immune environment for either tumor progression or an immune-modulatory effect.

*IL-10* expression was increased in PBMCs from all groups of CRC patients, showing 36-fold increase in expression, in average, compared to healthy donors. IL-10 is an inhibitory cytokine with anti-inflammatory as well as immunosuppressive properties. In the context of malignancy, its elevated levels in cancer patients can have both beneficial and detrimental effects. IL-10 can promote immune evasion by tumor cells by inhibiting the activation and function of effector T cells, NK cells, and macrophages [58]. Moreover, IL-10 stimulates the expression of PD-L1 on tumor cells [59, 60], thereby inhibiting T cells and NK cells through the activation of the PD-1/PD-L1 immune checkpoint signaling pathway. Esen et al. previously showed that PD-1^+^ NK cells have increased production of IL-10 [51]. The increased expression of *IL-10* by immune cells can also result from the release of various factors by tumor cells, creating an immunosuppressive microenvironment. Notably, IL-10 as an anti-inflammatory cytokine inhibits the production of the pro-inflammatory cytokines; IL-6 and TNF-α. This is consistent with our finding that IL-6 is downregulated in PBMCs of CRC patients. By inhibiting these inflammatory cytokines IL-10 inhibits the activation of antigen-presenting cells, such as dendritic cells and macrophages, which are crucial for initiating anti-tumor immune responses. High levels of IL-10 may promote the expansion of Tregs which suppress the immune response and contribute to immune tolerance towards tumor cells [58]. Chronic inflammation as a hallmark of cancer can be counteracted by IL-10 overexpression modulating excessive inflammation. On the other hand, IL-10 plays a key role in inhibiting tumor growth by recruiting and activating cytotoxic CD8^+^ T cells and NK cells, enhancing immune memory, suppressing pro-inflammatory M1 macrophages and Th17 cells, reducing pro-angiogenic factor synthesis, and limiting pro-inflammatory cytokine release that promotes tumor progression [61].

The protein encoded by the *CD27* gene belongs to the TNF-receptor superfamily and is essential for the development and long-term maintenance of T cell immunity. It interacts with its ligand CD70 on antigen-presenting cells (APCs) and plays a pivotal role in regulating B cell activation and immunoglobulin production. Research by Muth et al. [62] demonstrated that CD27 expression on regulatory T cells (Tregs) suppresses immune responses against tumors [62]. Additionally, Muth et al. [62] found that eliminating CD27 in Tregs works synergistically with PD-1 checkpoint inhibitors, enhancing cytotoxic T lymphocyte (CTL)-mediated immunity against solid tumors. Similarly, Calus et al. reported that CD27 signaling increases the frequency of regulatory T cells and promotes tumor growth [63]. CD27, is generally found on naive T and memory B and T cell populations and subsets of NK cells [64]. Within the tumor microenvironment, CD70-CD27 axis plays a crucial role in immune evasion aside from its impact on Treg cells. CD70 is aberrantly expressed on malignant cells in solid tumors. This aberrant CD70/CD27 interaction contributes to immune evasion by influencing the tumor microenvironment (TME) and promoting tumor progression [65]. Previously it was shown patients with advanced lung cancer has high serum level of CD27 which correlates with poor performance status and reduced survival suggesting the involvement of the CD70-CD27 axis [66]. CD70-CD27 signaling was shown to induce apoptosis and T cell exhaustion [64].

CD3G or CD3 γ subunit of T-cell receptor complex is the CD3-γ polypeptide, which together with CD3-ε, -δ and -ζ, and the T-cell receptor α/β and γ/δ heterodimers, forms the T-cell receptor-CD3 complex. Overexpression of *CD3G* in PBMCs isolated from CRC patients indicate increased T cell activation and can be explained by the chronic inflammation associated with CRC. However, the increased expression of CD3G in T cells of CRC patients can also indicate that these T-cells are exhausted or dysfunctional where they undergo a compensatory mechanism by upregulating *CD3G* expression. Previously it was shown that progenitor and terminally exhausted T cells have high expression of TCR-responsive transcription factor BATF which complexes with IRF4 to characterize exhaustion states [67]. BATF and IRF4 are both induced by TCR stimulation and induce the expression of many genes associated with T cell activation and differentiation [68]. Similarly, *CD8B* which is a crucial component of the CD8 co-receptor [69] is upregulated in PBMCs of CRC patients. Again, overexpression of CD8B reflects increased activation of, as well as exhausted or dysfunctional T cells. *CD2* which is s a co-receptor expressed on T cells that plays a role in T-cell activation and adhesion [70] is also upregulated in PBMCs of CRC patients reflecting increased activation of, as well as exhausted or dysfunctional T cells.

FAS (CD95), a member of the tumour necrosis factor (TNF) and nerve growth factor (NGF) family of receptors [71] is expressed on T cells, B cells and macrophages. With respect to B cells, it was previously shown that the interaction between CD40 with an activated T cell expressing CD154 (CD40L), causes up-regulation of CD95 expression on B cells and renders them susceptible to CD95-mediated apoptosis [72, 73]. For T cells, overexpression of *FAS* is an indication that T cells are undergoing activation induced cell death through Fas-mediated apoptosis [74]. Activation induced cell death is a mechanism activated when activated T cells undergo repetitive TCR stimulation [75].

Notably, upregulation of exhaustion markers like IL10, LAG3, CD27, FAS, CD8B occur in early-stage CRC (stage II) as well as late stages (stage III and stage IV) indicating that immune suppression mechanisms exist even in early stages of CRC. This is consistent with the “immune editing” paradigm where immune activation and suppression coexist in early-stage tumors [76]. This challenges the notion that immune dysfunction emerges only in late-stage CRC; instead, in stage II compensatory suppression begins (IL10, LAG3, CD27, FAS, CD8B upregulation), potentially priming the microenvironment for progression. The persistence of these signatures in stages III–IV suggests progressive dysfunction rather than de novo exhaustion. Our findings imply that immunotherapies targeting checkpoint inhibitors (e.g., anti-LAG3) or Th2 skewing (CCR3/IL5) could be beneficial even in stage II, before irreversible exhaustion dominates. Future studies should validate whether these transcriptomic shifts predict therapeutic response in early-stage patients or represent tumor-intrinsic evasion mechanisms or host-derived compensatory responses.

Interestingly, several of the genes upregulated in CRC PBMCs—including IL-10, LAG3, and CD27—are also associated with chronic viral infections such as HIV and hepatitis B/C, which are known to induce immune exhaustion and T cell dysfunction. In both cancer and chronic infection, prolonged antigen exposure drives the upregulation of inhibitory molecules and regulatory cytokines, contributing to T cell exhaustion and suppression of effector functions [77]. For instance, IL-10 is a key immunosuppressive cytokine elevated in chronic infections and tumors alike, dampening APC and T cell activity [78]. LAG3, a well-characterized marker of exhausted T cells and NK cells in chronic viral infections, also showed increased expression in our study, supporting the notion of an exhausted immune phenotype in CRC [79]. CD27 is also implicated in chronic viral infections such as HIV, where its persistent expression on T cells is associated with immune activation and eventual T cell dysfunction, reflecting features of exhaustion similar to those observed in cancer [80]. These parallels suggest that the immune dysregulation observed in CRC may mirror persistent infection-like immune suppression, with implications for therapeutic strategies aimed at reversing immune exhaustion. Additional genes such as FAS, IL18R1, and CD8B, which were upregulated in CRC PBMCs, are also implicated in chronic viral infections, where their persistent activation contributes to immune cell exhaustion, apoptosis, and functional impairment. Similarly, TLR1 and TLR6 are often dysregulated in long-term infections, supporting the idea that CRC induces a systemic immune landscape resembling chronic inflammation associated with persistent infection [81, 82].

In our gene expression profiling, we chose the pooling approach of RNA samples to allow for focus on group-level trends and reduce variability introduced by individual biological differences. Previous studies obtained similar results on gene expression profiling from pooling RNA samples compared to individual RNA samples before RNA sequencing or microarray, concluding that pooled RNA samples can be used to identify differentially expressed genes in a manner similar to analyzing individual RNA samples [83–87]. However, we recognize that this method limits our ability to evaluate interpatient heterogeneity. In fact, analyses at the individual sample level would complement pooled data. This approach would provide a more comprehensive understanding of interpatient heterogeneity and its role in immune dysfunction, which is indeed a crucial aspect of this research area. Therefore, we acknowledge the pooling approach as a limitation of our study.

While we observed non-statistical difference in serum IgG subclasses in CRC compared to healthy donors, one of the limitations of our study lies in the use of ELISA to measure total IgG and its subclasses in the serum of CRC patients. While this method provides an overview of the humoral immune response, it lacks the ability to distinguish antigen-specific IgG responses. The absence of antigen specificity limits our ability to directly correlate the observed IgG levels with tumor-associated antigens or CRC-specific immune responses. ELISA-based measurement of total IgG subclasses reflects systemic immunoglobulin production but does not account for variations in antigen-specific immune activity that might offer deeper insights into the immune-tumor interaction. Antigen-specific IgG profiles, particularly against CRC-related antigens, could provide a more detailed understanding of the role of the humoral immune response in CRC. Future studies incorporating techniques such as flow cytometry or antigen-specific ELISA using tumor-derived antigens would address this limitation, offering more precise insights into the humoral immune response specific to CRC.

While previous studies, such as those by Shaath et al. [8], Nichita et al. [9], and Ciarloni et al. [10], have broadly characterized systemic immune alterations in CRC using transcriptomic approaches, our study provides a targeted analysis of T and B cell activation genes, offering mechanistic insights into adaptive immune dysfunction. Unlike prior work, which primarily identified global gene signatures, we focused on a focused panel of 84 genes implicated in T and B cell signaling, revealing specific dysregulations (e.g., *CCR3, LAG3, IL10*) that may drive immune evasion and exhaustion in CRC. Notably, our discovery of *CCL3* downregulation and *IL18R1* upregulation in PBMCs highlights novel systemic immune dysregulation not previously linked to CRC progression. Furthermore, our integration of IgG subclass profiling (though not statistically significant) adds a humoral immunity dimension absent in earlier studies. By bridging cellular and humoral immune responses, our work not only supports prior findings (e.g., elevated IL10 [8]) but also identifies actionable targets (e.g., LAG3 for checkpoint inhibition or Th2 skewing (CCR3/IL5)) in early as well as late stages of CRC. This refined understanding of adaptive immune dysregulation could inform personalized immunotherapies for CRC patients.

## Conclusion

Out of the 84 genes responsible for T and B-cell activation, a total of 18 genes were differentially expressed in PBMCs isolated from CRC patients. Thirteen genes were upregulated (*IL18R1, CCR3, IL10, IL5, TLR6, LAG3, TLR1, MAP3K7, CD27, CD8B, FAS, CD2* and *CD3G*) and 5 genes were downregulated (*IL6*, *CCL3, CSF2, CXCR3, and TNFSF14*). Results showed a non-significant change in IgG1, IgG2, IgG3 and IgG4 subclasses across all CRC subtypes. Our findings provide insights into the adaptive immune dysfunction in CRC, offering a detailed profile of gene expression changes associated with T and B cell activation and antibody production. Understanding these dysregulations may enhance the development of targeted immunotherapies, potentially improving outcomes for CRC patients through more personalized immunomodulatory approaches.

## Supplementary Information

Supplementary material 1.

## Data Availability

All data on the measured T cell and B cell activation genes differential expression in PCMCs and serum IgG subclasses in CRC patients that support the findings of this study are included within this paper.

## Declarations

### Informed consents

Informed consents were obtained from CRC patients and healthy donors in accordance with the Declaration of Helsinki.
