# Supplementary material for: Adaptive immune changes in colorectal cancer: a focus on T and B cell activation genes
Source: Discov Oncol. 2025 Jun 8;16:1032. doi: 10.1007/s12672-025-02794-8 (PMC12146245; doi:10.1007/s12672-025-02794-8)
Supplement: Supplementary file 1 — Supplementary material 1. [file 12672_2025_2794_MOESM1_ESM.docx]

Supplementary Figure 1 shows a scatter plot comparing the average fold regulation of 84 genes between the two groups, with red dots indicating upregulated genes and green dots indicating downregulated genes. The scatter plot was generated using the GeneGlobe data analysis tool (Qiagen).


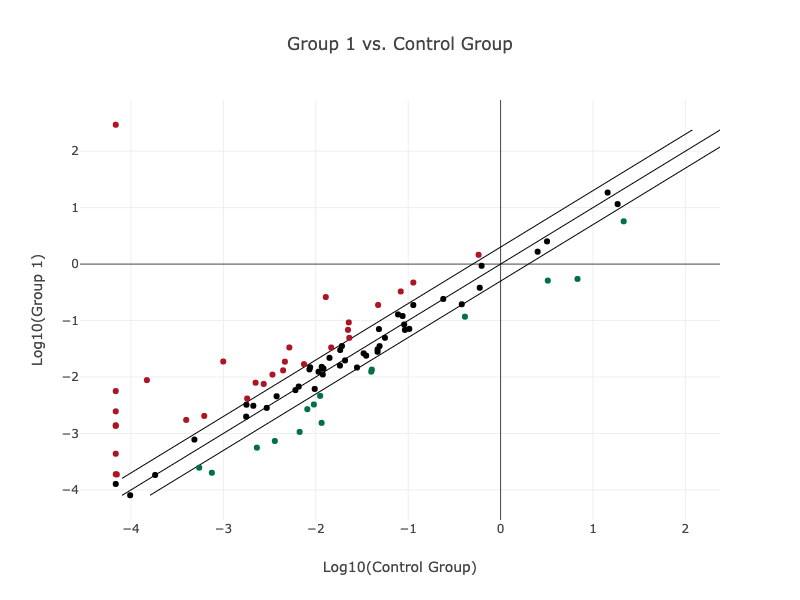


**Supplementary Figure 1: Scatter plot comparing the average fold regulation of the 84 T and B cell activation genes in CRC patients compared to healthy donors. The red and green dots represent up-regulated and down-regulated genes, respectively. The scatter plot was created by the GeneGlobe data analysis webtool provided by Qiagen and available at https://geneglobe.qiagen.com/eg/analyze.**

Supplementary Figures 2 and 3 show gene interaction network of downregulated and upregulated genes, respectively in PBMCs from patients with CRC identified using GeneMANIA tool.


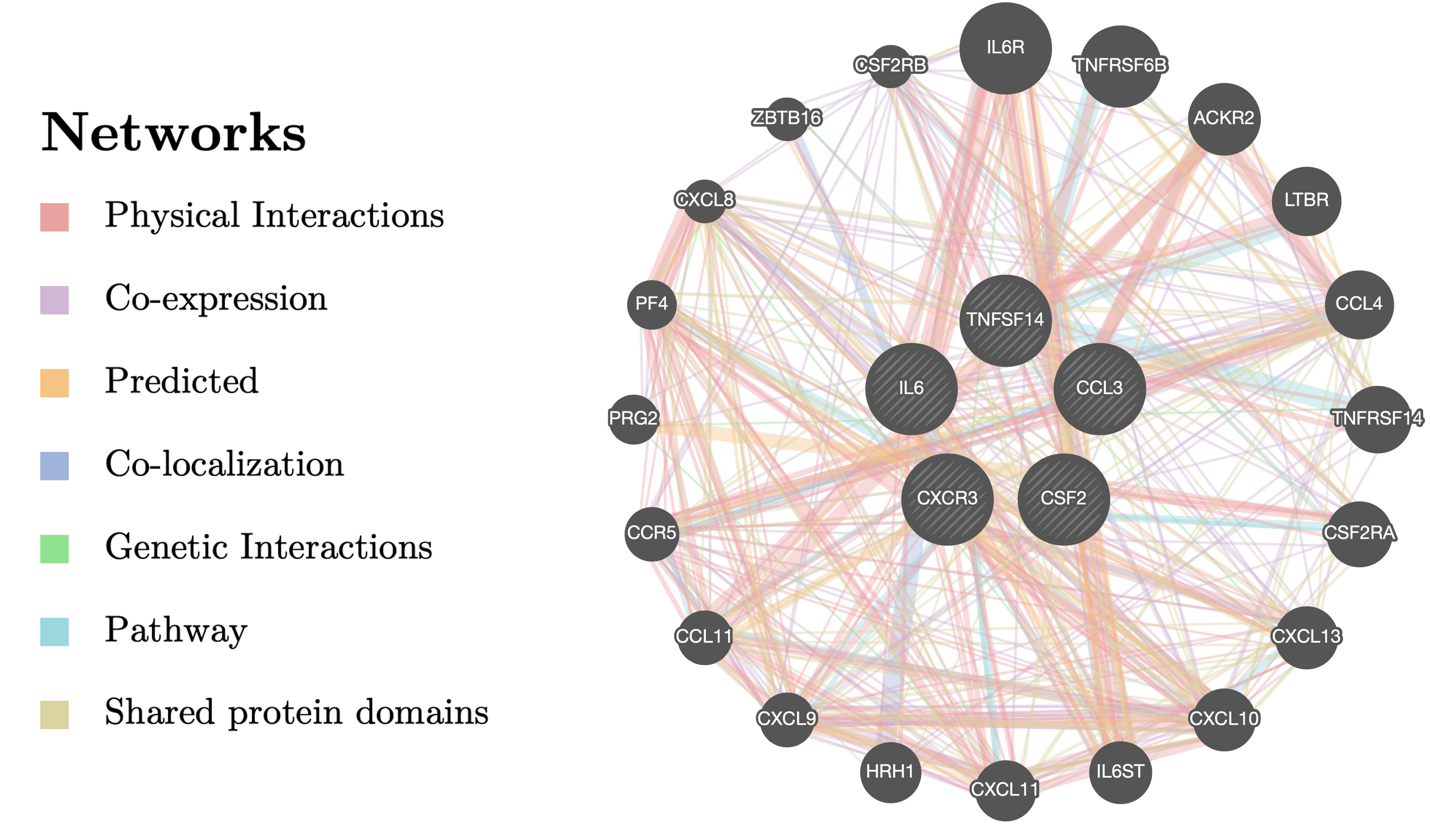


**Supplementary Figure 2:** **Gene interaction network of downregulated genes in PBMCs from patients with CRC identified using GeneMANIA. Striped nodes represent downregulated genes (5), while solid nodes represent related genes (20) and 494 total links. Edges indicate functional associations, including physical interactions, co-expression, and shared pathways. The network was generated using GeneMANIA, Application version : 3.6.0 with a prediction confidence score of 0.6 [1]**

**
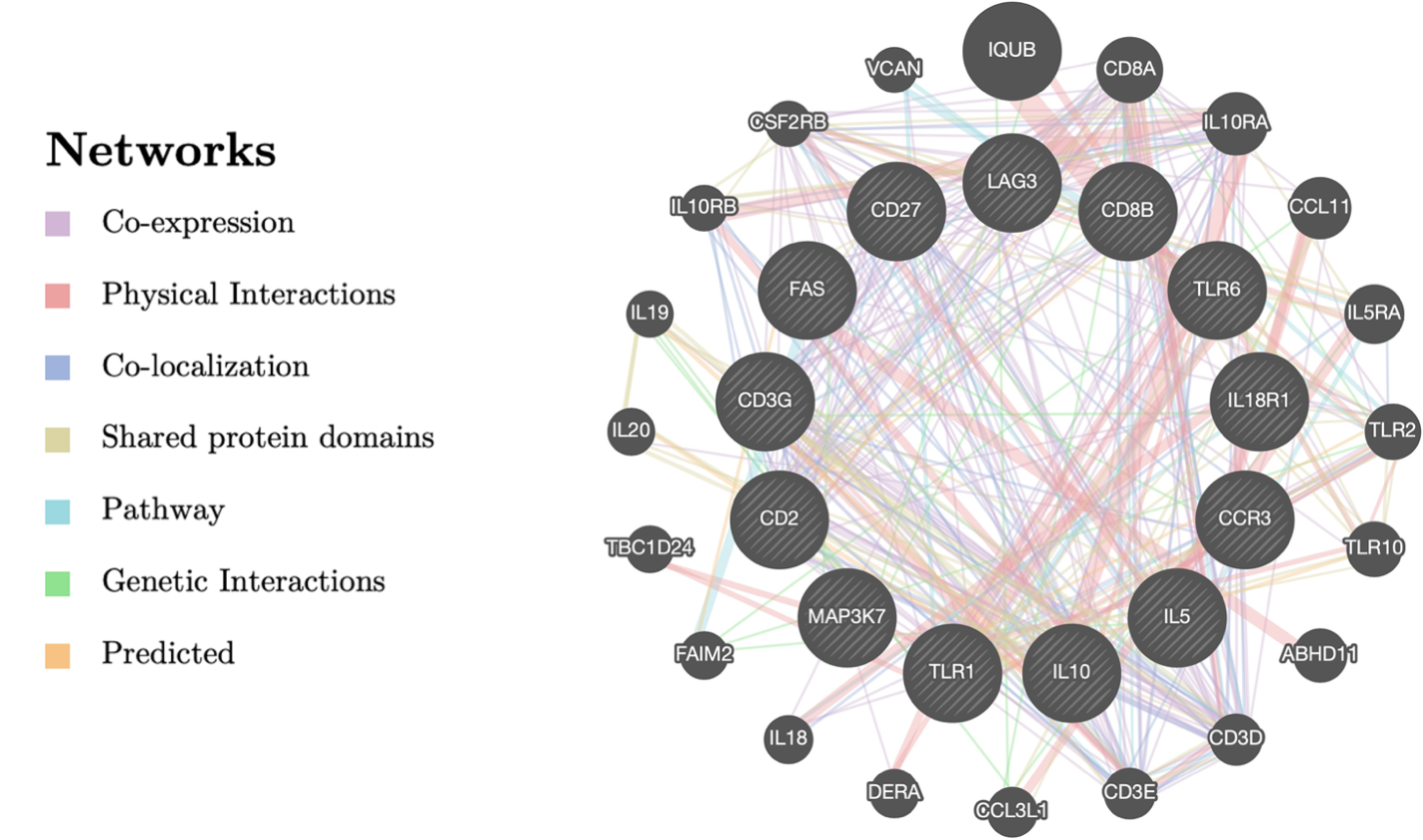
**

**Supplementary Figure 3: Gene interaction network of upregulated genes in PBMCs from patients with CRC identified using GeneMANIA. Striped nodes represent upregulated genes (13), while solid nodes represent related genes (20) and 388 total links. Edges indicate functional associations, including physical interactions, co-expression, and shared pathways. The network was generated using GeneMANIA, Application version : 3.6.0 with a prediction confidence score of 0.6 [1].**

**References:**

[1] D. Warde-Farley *et al.*, “The GeneMANIA prediction server: biological network integration for gene prioritization and predicting gene function,” *Nucleic Acids Res.*, vol. 38, no. suppl_2, pp. W214–W220, Jul. 2010, doi: 10.1093/nar/gkq537.
